# Iodoform as a Vermifuge

**Published:** 1881-08

**Authors:** F. L. Sim

**Affiliations:** Professor of Obstetrics and Diseases of Children in Memphis Hospital Medical College


### Selections.

### IODOFORM AS A VERMIFUGE.

By F. L. SIM, M.D.,

Professor of Obstetrics and Diseases of Children in Memphis Hospital Medical College.

During April, 1876, Mr. B., a robust, healthy young man, æt. twenty-five, an engineer by occupation, by birth an Italian, but a Memphian from childhood, consulted me for treatment. He complained of intolerance of light and sound, with great debility and nervous prostration. His digestion was markedly impaired, stomach becoming oppressively distended after each meal, and in consequence was compelled to indulge in the most simple diet, viz. oatmeal, tapioca, well-boiled rice, milk, etc. A sense of hunger, extremely distressing, often causing faintness, was constantly felt, even immediately after a repast.

His sleep was disturbed and nervous, sometimes talking and having strange visions during the entire night, so that it was never refreshing.

A visit to Hot Springs, Ark., during 1878, greatly improved his health, but upon returning soon relapsed into his former condition.

May 1st, 1880, during my absence, he called upon another physician, on account of disease of the prostate, for which he was advised to take one grain of iodoform three times a day.

After continuing this treatment for some time, he observed some segments of tape-worm in his stools.

Upon again consulting the physician, was advised to take santonine in fifteen grain doses. This vomited him so severely that its use was abandoned.

Upon my return I advised half drachm of oil male fern, to be followed in four hours by a large dose of castor oil and turpentine.

This caused the expulsion of eighteen feet of the worm. During the succeeding two or three weeks the patient greatly improved. At the end of this time he again had recourse to the iodoform for relief of uneasiness about the neck of the bladder.

Three days later segments of the worm were noticed; this continued as long as the pills were taken regularly; when they were discontinued the proglottides ceased to pass.

October, 1880, I again advised the male fern as before, this time resulting in the expulsion of thirty feet.

Although no head was discovered, we hoped the entire worm had been passed.

The health of the patient continued to improve for about one month, when the former symptoms again made their appearance.

The iodoform was this time resorted to for diagnostic purposes, and after a few days' use of it, segments of the worm were found in the stools. Its use was persisted in for several days before adopting curative treatment, with the view of sickening the worm, thereby rendering its complete expulsion more certain.

Early in January, 1881, a large dose of kusso was taken, but at once rejected by the stomach.

A drachm of oil of male fern (double the former dose) was then taken, followed by a brisk cathartic four hours later.

This resulted in the expulsion of twenty-one feet, and probably all of the tape-worm, as the iodoform has been taken several times since without discovering it. The health of the patient is entirely restored, complains of nothing, eats heartily and his digestion is perfectly good.

Few conditions are more difficult to diagnose than the presence of a tape-worm in the alimentary canal; in fact it is impossible to arrive at a definite conclusion without seeing portions of the worm. It is however of great importance to have positive information not only as to the existence of the worm, but also as to the variety, before instituting expulsive treatment.

Heller says upon this subject: "The diagnosis is only certain when it is known that portions of the worm have been expelled. The diagnosis of the variety of worm is indispensable. For otherwise, if a tape-worm which has a great power of resistance were present, we might not attack it with sufficient energy, and thus the patient would have to suffer a repetition of his torture, or if a variety were present that was easy to expel, we might act in the most energetic manner without any sufficient reason."

Although the symptoms attending tape-worm are very indefinite, yet they are often sufficiently well marked to lead us to *suspect* its existence, and in such cases the iodoform would prove invaluable as a diagnostic agent.

The preparatory treatment always recommended by authors, such as eating onions, garlic, salt herring, etc., the day before the adoption of curative treatment, contemplates the sickening of the worm, that it may fall an easy prey to the plan adopted for its expulsion.

The iodoform would certainly be less objectionable, and judging from the above experience, much more potent, for this purpose.

Iodoform is certainly not a vermiciticide, as the segments were expelled alive. How it acts, I will not undertake to state, but think it quite probable that the odor proves intolerable and actually "stinks him out."

February 12th, 1881, Mr. S—— called for advice.

He complained of intolerable tickling and itching about the rectum and anus, becoming greatly aggravated after going to bed at night.

This distressing feeling had annoyed him for months; recently, however, it had grown so violent that he was compelled to seek relief.

I ordered a suppository of cocoa butter containing seven grains of iodoform, to be introduced into the rectum at bed-time.

The following morning he reported having passed "a handful of needle worms" (oxyuris vermicularis.)

Complete relief was obtained in about one hour after the introduction of the suppository, and the first good night's rest enjoyed for many nights followed.

I ordered one grain of iodoform taken by the mouth three times a day, the suppositories to be repeated if itching returned.

No suppositories were subsequently indicated. After a continuation of the iodoform four days the worms began to pass, and were found in every stool for six days.

No evidence of their presence has since existed.

In two other instances during the administration of iodoform to adults for other purposes, a number of the ascar lumbricoid were ejected.—*Miss. Valley Med. Monthly.*

